# Preoperative C-reactive protein-to-albumin ratio as a prognostic marker of histopathological characteristics and survival outcomes following radical cystectomy

**DOI:** 10.3389/fonc.2026.1895361

**Published:** 2026-07-28

**Authors:** Nebojša Prijović, Miodrag Aćimović, Uroš Babić, Veljko Šantrić, Branko Stanković, Bojan Čegar, Jelena Ilić Živojinović, Ivan Vuković

**Affiliations:** 1 Clinic of Urology, University Clinical Center of Serbia, Belgrade, Serbia; 2 Faculty of Medicine, University of Belgrade, Belgrade, Serbia; 3 Institute of Hygiene and Medical Ecology, Faculty of Medicine, University of Belgrade, Belgrade, Serbia

**Keywords:** bladder cancer, C-reactive protein-to-albumin ratio, immunonutrition, inflammation, radical cystectomy

## Abstract

**Introduction:**

Bladder cancer represents the second most common urological malignancy in Europe, and radical cystectomy is the cornerstone of treatment for muscle-invasive bladder cancer. Current research highlights the prognostic significance of markers reflecting inflammatory and nutritional status in oncology. As such, the aim of our study was to investigate the prognostic value of the C-reactive protein-to-albumin ratio (CAR) for histopathological characteristics and survival in bladder cancer patients who underwent radical cystectomy.

**Methods:**

A retrospective cohort study was conducted comprising 491 patients treated with radical cystectomy between 2017 and 2021, with postoperative survival follow up in June 2022.

**Results:**

Our results demonstrate that CAR is statistically significantly higher in patients with non-urothelial carcinoma compared to patients with urothelial carcinoma in the presence of lymphovascular invasion and cases of positive lymph nodes. CAR directly correlates with the pathological tumor stage. Multivariate analysis revealed that alongside histopathological tumor characteristics, CAR and age are independent predictors of patient survival following radical cystectomy.

**Discussion:**

Our study demonstrated a multifaceted association between CAR and the histopathological characteristics of bladder cancer following radical cystectomy. Furthermore, it was shown that CAR is an independent predictor of survival in these patients.

## 1 Introduction

Bladder cancer represents the second most common urological malignancy in the Republic of Serbia, as well as Europe (1, 2). According to data from the Institute of Public Health of Serbia Dr. Milan Jovanović Batut, bladder cancer was the sixth most frequently diagnosed malignancy and the seventh leading cause of cancer-related mortality in the Republic of Serbia in 2022 (1), with approximately 2,300 new cases and 700 deaths. As the fourth most frequent malignancy within the male population in Serbia, the incidence of bladder cancer in men is over twice as high as in women (1). In Europe, this cancer represents the fifth most common malignancy, with over 220,000 new cases in 2022, while in terms of mortality, it ranks eighth, with approximately 70,000 deaths in the same year (2). These figures underscore the significant public health and socioeconomic impact of bladder cancer detection and treatment.

Over 90% of bladder cancer cases are histologically classified as urothelial carcinoma (3). The presence of tumor invasion into the muscular layer of the bladder wall determines whether it is non-muscle-invasive bladder cancer (NMIBC) or muscle-invasive bladder cancer (MIBC) (4). At diagnosis, approximately 70% of patients present with NMIBC, which may progress to MIBC in up to 50% of cases over time (5, 6). The cornerstone of NMIBC management is transurethral resection of the tumor (TURBT), alongside the prevention of recurrence and progression (7). Given that approximately 85% of patients with untreated MIBC die due to the disease within two years (8), radical cystectomy with urinary diversion remains the standard of care for patients with MIBC and high-risk NMIBC (7).

In modern oncology, inflammation is considered a hallmark of cancer, facilitating tumor growth and progression (9). Hence, recent research has focused on various inflammatory parameters and indices as potential biomarkers for diverse malignancies (10–12) such as bladder cancer (13, 14). In addition to inflammation, it has been observed that nutritional status and its indicators influence health outcomes in oncological patients (15).

Among the most frequently utilized inflammatory parameters in clinical practice is C-reactive protein (CRP). As an acute-phase protein synthesized by the liver, CRP is routinely used to assess the degree of inflammation primarily in infections (16), but also in other pro-inflammatory states such as autoimmune processes (17) and malignancies (18). Its significance as an oncological biomarker has been extensively investigated in recent years (19). Albumin is also a synthetic product of the liver, and serves as an indicator of a patient’s nutritional status (20). Current studies are analyzing a relatively novel immuno-nutritional parameter, the C-reactive protein-to-albumin ratio (CAR), as a potential biomarker of various malignancies (21). However, the significance of this parameter remains insufficiently explored in bladder cancer patients, particularly regarding its association with histopathological tumor characteristics.

Given that our previous research suggested a correlation between other inflammatory and nutritional markers, and the histopathological characteristics of bladder cancer patients treated with radical cystectomy, while few studies on a relatively small number of patients have investigated CAR in this population, the aim of our study was to evaluate the association between the C-reactive protein-to-albumin ratio and histopathological features and survival in patients undergoing radical cystectomy in a larger sample of patients.

## 2 Materials and methods

### 2.1 Study cohort and baseline characteristics

A retrospective study was conducted at the Clinic of Urology, University Clinical Center of Serbia, including all patients who underwent radical cystectomy for primary bladder cancer between January 1, 2017, and December 31, 2021. Data were extracted from the same database utilized in a previously published studies, where the methodology is described in detail (13, 14).

The study included all patients who underwent radical cystectomy with histopathologically verified primary muscle-invasive bladder cancer (clinical stage cT2-T4 N0 M0) or high-risk/unresectable non-muscle-invasive bladder cancer, provided there was no preoperative imaging evidence of metastatic disease. Patients with hematological disorders or immunodeficiency conditions that could interfere with the hematological and biochemical parameters of interest were excluded. Laboratory analyses of preoperative blood samples were performed at the Center for Medical Biochemistry, University Clinical Center of Serbia. For the purposes of this study, serum levels of C-reactive protein (CRP) and albumin were collected to calculate the CRP-to-albumin ratio (CAR). The histopathological diagnosis and pathological stage of the specimens following radical cystectomy were determined according to the current World Health Organization (WHO) classification and the TNM staging system by an experienced uropathologist (22, 23). The histopathological characteristics of the patients were based on the analysis of the radical cystectomy specimens. Patient follow up was maintained until June 30, 2022, ensuring a minimum follow-up period of six months for the final patient enrolled. Overall survival data were obtained during follow-up examinations or via telephone contact. Overall survival was defined as the time, in months, from surgery to death from any cause for deceased patients, or until June 30, 2022 for patients who were alive.

### 2.2 Statistical analysis

Descriptive and analytical statistical methods were employed for data analysis. Given that the CRP, albumin, and CAR values did not follow normal distribution, the Mann–Whitney U-test was used to assess differences between the examined patient groups, while Spearman’s rank correlation was used to evaluate correlations. A Cox proportional hazard model was used to identify predictors of survival. In order to identify predictors of survival, we created overall prognostic models. In the univariate analysis, we included clinical-demographic characteristics (age, gender, Eastern Cooperative Oncology Group (ECOG) performance status, American Society of Anesthesiologists (ASA) score, chronic renal failure, neoadjuvant chemotherapy), operative characteristics (performance of pelvic lymphadenectomy, number of excised lymph nodes), histopathological characteristics (pathological stage, presence of lymphovascular invasion, status of lymph nodes, status of surgical margins, presence of variant histologies), as well as CAR and log-transformed CAR. The parameters that were identified as significant predictors in the univariate analysis were included in the multivariate analysis. In order to evaluate CAR and logCAR as predictors of survival, we determined Harrell’s C-index and performed the Likelihood ratio test.

## 3 Results

Between 2017 and 2021, 491 patients meeting the inclusion criteria underwent radical cystectomy. The cohort comprised 387 (78.8%) male patients, with a median age of 67 years. The median C-reactive protein-to-albumin ratio (CAR) was 0.12 (IQR 0.05-0.51). On June 30, 2022, 269 (54.8%) patients were alive, with a median follow-up time of 18 months (IQR 10–36 months). Detailed sociodemographic, clinical, and laboratory characteristics are presented in **Table 1**.

**Table 1:** The sociodemographic, clinical, and laboratory characteristics of patients who underwent radical cystectomy.

Parameter	Total
Total, n	491
Sex, n (%)	
Male	387 (78.8)
Female	104 (21.2)
Age, median (IQR)	67.0 (62.0–72.0)
ECOG performance status, n (%)	
0	202 (41.1)
≥1	289 (58.9)
ASA, n (%)	
ASA ≤ 2	348 (70.9)
ASA>2	143 (29.1)
Chronic kidney disease, n(%)	
No	444 (90.4)
Yes	47 (9.6)
Neoadjuvant chemotherapy, n (%)	
No	425 (86.6)
Yes	66 (13.4)
Pelvic lymphadenectomy,n (%)	
No	144 (29.3)
Yes	347 (70.7)
Number of excised lymph nodes, median (IQR)	8 (4-13)
C-reactive protein (mg/L), mean ± SD	20.36 ± 42.19
Albumin (g/L), mean ± SD	39.44 ± 4.61
CRP/albumin ratio (CAR), median (IQR)	0.12 (0.05-0.51)
Status, n (%)	
Alive	269 (54.8)
Deceased	222 (45.2)
Follow-up time (months), median (IQR)	18 (10–36)
Alive	27 (14-44)
Deceased	11 (6-22)

IQR—interquartile range; ECOG—Eastern Cooperative Oncology Group; ASA— American Society of Anesthesiologists; SD—standard deviation.

Among the patients examined, urothelial carcinoma was the most prevalent histological type, identified in 459 (96.0%) cases. In 13 patients, the type of malignancy was not determined because the stage of the disease was assessed as pT0. Regarding the pathological stage of the disease, pT2 was the most frequent (32.6%), followed by pT3 (31.4%). Lymphovascular invasion was present in 336 (70.3%) patients, while lymph node metastases were histopathologically confirmed in 81 (23.3%) patients. **Table 2** provides a detailed overview of the histopathological characteristics of the cohort.

**Table 2:** The histopathological characteristics of patients following radical cystectomy.

Parameter	Total
Histological type, n (%)*	
Urothelial carcinoma	459 (96.0)
Non-urothelial carcinoma	19 (4.0)
Adenocarcinoma	8 (1.6)
Squamous cell carcinoma	7 (1.4)
Neuroendocrine carcinoma	3 (0.6)
Extragastrointestinal stromal tumor	1 (0.2)
Variant histology, n (%)**	
Absent	369 (78.4)
Present	90 (19.6)
pT stage, n (%)	
pT0	13 (2.6)
pTa	3 (0.6)
pTis	9 (1.8)
pT1	37 (7.5)
pT2	160 (32.6)
pT3	154 (31.4)
pT4	115 (23.4)
Lymphovascular invasion, n (%)*	
Absent	142 (29.7)
Present	336 (70.3)
Surgical margin, n(%)	
Negative	415 (84.5)
Positive	76 (15.5)
Lymph nodes, n (%)***	
Negative	266 (76.7)
Positive	81 (23.3)

*The total is not 491 (100%) as no tumor was detected in the specimens of 13 patients (i.e., stage pT0).

**The total is not 491 (100%) because variant histologies only refer to urothelial carcinoma, whereby we do not include 13 patients with pT0 stage and 19 patients with Non-urothelial carcinoma.

***The total is not 491 (100%) because pelvic lymphadenectomy was performed in 347 patients.

**Table 3** presents comparison of the CRP-to-albumin ratio (CAR) across various histopathological characteristics, including histological type, pT stage, lymphovascular invasion (LVI), and lymph node status. CAR was statistically significantly higher in patients with non-urothelial carcinoma compared to those with urothelial carcinoma (0.37 (0.21-1.68) vs 0.12 (0.05-0.49), p = 0.003). Additionally, the ratio was significantly higher in the presence of lymphovascular invasion (0.18 (0.06-0.74) vs. 0.09 (0.03-0.24), p <0.001), as well as in cases with positive lymph nodes (0.25 (0.08-0.78) vs. 0.09 (0.04-0.34), p = 0.001).

**Table 3:** Comparison of the CRP-to-albumin ratio according to histopathological features in patients undergoing radical cystectomy.

Parameter	CAR (median (IQR))	p-value
Histological type		
Urothelial carcinoma	0.12 (0.05-0.49)	**0.003^a^**
Non-urothelial carcinoma	0.37 (0.21-1.68)	
pT stage		
NMIBC (pTa+pTis+pT1)	0.10 (0.04-0.24)	0.055^a^
MIBC (pT2–pT4)	0.15 (0.05-0.58)	
Lymphovascular invasion		
Absent	0.09 (0.03-0.24)	**p< 0.001^a^**
Present	0.18 (0.06-0.74)	
Lymph node status		
Negative	0.09 (0.04-0.34)	**0.001^a^**
Positive	0.25 (0.08-0.78)	

^a^ Mann–Whitney U-test.Bolded values are statistically significant.

Spearman’s rank correlation demonstrated a statistically significant direct correlation between the CRP-to-albumin ratio (CAR) and the pathological tumor stage (p < 0.001), presented in **Table 4**.

**Table 4:** Spearman’s rank correlation between the CRP-to-albumin ratio (CAR) and pathological tumor stage.

Parameter	Correlation coefficient	p-value
CAR	0.264	**<0.001^a^**

^a^ Spearman’s rank correlation coefficient.Bolded values are statistically significant.

To identify predictors of survival in patients undergoing radical cystectomy, a overall prognostic model was constructed incorporating clinico-demographic characteristics, operative characteristics, histopathological characteristics, as well as CAR and log-transformed CAR. The parameters that were identified as significant predictors in the univariate analysis were included in the multivariate analysis. In the multivariate analysis, age, pT stage, LVI, LN status and logCAR (HR 1.335, 95% CI 1.022-1.743, p=0.034) were identified as independent predictors. Multivariate analysis included 300 patients with complete data on all parameters included in the prognostic model (the number of included patients was mostly limited by the number of those who underwent pelvic lymphadenectomy and whose lymph node status was known). A detailed presentation of the univariate and multivariate Cox regression analysis of survival is provided in **Table 5**. Models are created for age, pT stadium, LVI and LN and logCAR (model with and model without). The Likelihood ratio test for model 1 (without logCAR) is 59.14 (p<0.001) and for model 2 (with logCAR) is 63.76 (p<0.001). The difference between models is 4.622 with DF = 1 and p=0.032 which suggests that the adding CAR significantly improves the model. C index for model 1 is 0.694 and 0. 709 for model 2. The estimated difference is 0.016 with z-score=2.067 and p=0.038 which also confirms that adding logCAR significantly improves the model.

**Table 5:** Death outcome, descriptive statistics, univariable and multivariable cox regression of survival predictors in patients who underwent radical cystectomy.

Parameter	Death outcome		Univariable		Multivariable^b^	
	No	Yes	HR (95% CI)	p value	HR (95% CI)	p value
Age (mean ± SD)	64.9 ± 8.2	67.6 ± 7.6	1.032 (1.014-1.050)	**<0.001**	1.034 (1.012-1.056)	**0.002**
Sex, n(%)			1.027 (0.737-1.431)	0.875		
Male	210 (54.3)	177 (45.7)				
Female	59 (56.7)	45 (43.3)				
ECOG, n(%)			1.388(1.048-1.838)	**0.022**		
0	124 (61.4)	78 (38.6)				
≥1	145 (50.2)	144 (49.8)				
pT stage, n(%)			1.672 (1.447-1.932)	**<0.001**	1.404 (1.109-1.778)	**0.005**
T0	11 (84.6)	2 (15.4)				
T1	40 (81.6)	9 (18.4)				
T2	112 (70.0)	48 (30.0)				
T3	67 (43.5)	87 (56.5)				
T4	39 (33.9)	76 (66.1)				
LVI, n(%)			2.621 (1.814-3.786)	**<0.001**	1.861 (1.003-3.453)	**0.049**
Present	152 (45.2)	184 (54.8)				
Absent	106 (74.6)	36 (25.4)				
LN status, n(%)			2.385 (1.703-3.339)	**<0.001**	1.633 (1.119-2.383)	**0.011**
Positive	25 (30.9)	56 (69.1)				
Negative	176 (66.2)	90 (33.8)				
Surgical margins, n(%)			1.882(1.362-2.603)	**<0.001**		
Positive	28 (36.8)	48 (63.2)				
Negative	241 (58.1)	174 (41.9)				
Variant histology, n(%)			1.526 (1.102-2.114)	**0.011**		
Present	42 (46.7)	48 (53.3)				
Absent	209 (56.6)	160 (43.4)				
CAR (mean ± SD)	0.088 ± 0.237	0.247 ± 0.762	1.220 (1.107-1.345)	**<0.001**		
LogCAR (mean ± SD)	-0.942 ± 0.618	-0.617 ± 0.725	1.635 (1.337-1.998)	**<0.001**	1.335 (1.022-1.743)	**0.034**
Neoadjuvant chemotherapy, n(%)			1.324 (0.904-1.938)	0.149		
Yes	34 (51.5)	32 (48.5)				
No	235 (55.3)	190 (44.7)				
ASA, n(%)			1.337 (1.008-1.774)	**0.044**		
ASA ≤ 2	203 (58.3)	145 (41.1)				
ASA>2	66 (46.2)	77 (53.8)				
Pelvic lymphadenectomy, n(%)			0.760 (0.572-1.008)	0.057		
Yes	201 (57.9)	146 (42.1)				
No	68 (47.2)	76 (52.8)				
LN excised, median (IQR)	9 (5-12)	7 (4-13)	1.001 (0.979-1.023)	0.945		
HBI, n(%)			1.285 (0.846-1.950)	0.239		
Yes	21 (44.7)	26 (55.3)				
No	248 (55.9)	196 (44.1)				

IQR, interquartile range; SD, standard deviation; HR, hazard ratio; CI, confidence interval.

ECOG, Eastern Cooperative Oncology Group; ASA, American Society of Anesthesiologists; LVI, lymphovascular invasion; LN, lymph node; CAR, C-reactive protein-to-albumin ratio; LogCAR, log transformed CAR.

^b^ Backward method for variable reduction.Bolded values are statistically significant.

## 4 Discussion

This study demonstrated the multifaceted association between the preoperative C-reactive protein-to-albumin ratio (CAR) and histopathological characteristics in patients with bladder cancer who underwent radical cystectomy. Furthermore, our findings identified CAR as an independent predictor of survival in this patient population. Histopathological characteristics remain the most critical prognostic factors for survival following radical cystectomy (24, 25). As such, identifying preoperative parameters associated with these characteristics may facilitate improved patient stratification, treatment strategies, and postoperative surveillance.

C-reactive protein (CRP) is an acute-phase protein synthesized by the liver, initially discovered as a protein that precipitates bacterial antigens (16, 26). Subsequent research has elucidated its multifaceted roles in the inflammatory process (16), extending beyond infection to autoimmune diseases and oncology (17–19). Contemporary cancer research underscores the role of inflammation in fostering a favorable microenvironment for tumor progression and metastasis (27, 28). Current studies also link CRP directly to tumor progression (29). Conversely, albumin, also synthesized by the liver, serves as a negative acute-phase protein and a reliable indicator of nutritional status (16, 30). While existing literature in the field of bladder cancer highlights the individual prognostic significance of CRP and albumin in patients who underwent radical cystectomy (19, 30), data remain sparse on the association between the relatively novel CAR, and histopathological characteristics and clinical outcomes.

Our research revealed a significant association between preoperative CAR values and the histopathological profiles of patients who underwent radical cystectomy. Notably higher CAR values were observed in rare histological variants compared to the more common urothelial carcinoma. Non-urothelial bladder cancers, such as adenocarcinoma and squamous cell carcinoma, frequently present as advanced and more aggressive diseases compared to urothelial carcinoma (31, 32). Based on our results, we can conclude that higher CAR values were associated with more adverse pathological characteristics. Tumor stage is a paramount prognostic factor in patients treated with radical cystectomy (24). Our results show a positive correlation between CAR and pathological tumor stage, further suggesting that higher values of this parameter are associated with advanced disease. These observations align with the 2021 study by Zhang et al., which, in a cohort of 127 patients treated with radical cystectomy, indicated a correlation between CAR and pathological T stage (33).

Lymphovascular invasion (LVI) is also among the most significant prognostic factors in patients underwent radical cystectomy (25). LVI involves the presence of tumor cells within lymphatic vessels and vascular walls, and is recognized as a risk factor for occult metastases across various malignancies (34–36). Our study demonstrated significantly higher CAR values in the presence of this poor prognostic factor in radical cystectomy specimens. Given that, to our knowledge, the association between CAR and LVI in bladder cancer has not been previously investigated, we interpreted our results within the context of other malignancies. Chen et al., in a study of 406 patients with clear cell renal cell carcinoma, identified a correlation between higher CAR values and the presence of LVI (37).

Another critical prognostic factor following radical cystectomy is lymph node status (24). The presence of metastases in resected lymph nodes is associated with diminished survival (24). Despite the routine use of modern imaging, preoperative lymph node status cannot yet be reliably predicted (38). Therefore, identifying preoperative predictors of nodal metastasis is of great clinical importance. Our previous research on bladder cancer demonstrated the potential predictive value of nutritional status markers for lymph node metastasis, particularly the Geriatric Nutritional Risk Index (GNRI) (13). The results of the current study show that CAR values are significantly higher in cases where metastases are present in resected pelvic lymph nodes. Our observations in this cohort of a larger number of patients confirm the findings of Zhang et al., who showed an association between elevated CAR values and positive lymph node status in 127 patients (33). These findings suggest that CAR may possess greater clinicopathological significance than CRP alone, as a 2025 meta-analysis by Feng et al. failed to demonstrate a significant association between CRP values and histopathological characteristics in bladder cancer (19).

In recent years, CAR has been investigated as a prognostic factor in various malignancies (37, 39, 40) such as bladder cancer (41). Our results show that in a multivariate model including established prognostic factors, preoperative CAR is an independent predictor of overall survival following radical cystectomy. Adding CAR to our prognostic model with proven histopathological prognostic factors statistically significantly, but modestly, improves this model. Our findings are consistent with two previous studies identifying preoperative CAR as a predictor of overall survival; however, those studies (from 2018 and 2021) involved smaller cohorts of 131 and 127 patients, respectively (33, 41). Research by Kuroda et al. in 2020 comprising 102 patients further highlighted that postoperatively elevated CAR values are associated with poorer cancer-specific survival (42). Beyond survival, CAR has also been identified as a potential prognostic marker of acute kidney injury following radical cystectomy (43).

Our study has several limitations. Specifically, this is a retrospective observational single-center study conducted on a relatively small number of patients with a comparatively short postoperative follow-up period. Given the retrospective nature of the study and the impossibility of obtaining reliable data on the postoperative course of the disease, it was not possible to determine cancer-specific survival and recurrence-free survival, which represents a significant limitation of our study. There is also an important limitation that may affect the potential generalization of our results, given the different characteristics of patients in other populations, as well as differences in preoperative treatment and available treatment methods in other health care institutions.

## 5 Conclusion

Our study demonstrated a multifaceted association between the C-reactive protein-to-albumin ratio (CAR) and the histopathological characteristics of bladder cancer following radical cystectomy. Significantly higher CAR values were observed in patients with non-urothelial bladder carcinoma in the presence of lymphovascular invasion and in cases with metastases in resected lymph nodes. Furthermore, CAR values positively correlate with the pathological tumor stage. Multivariate analysis identified age and CAR as independent predictors of survival, alongside established histopathological tumor characteristics.

This study evaluated the laboratory parameters CRP and albumin, which are routinely analyzed in daily clinical practice. The potential prognostic significance of CAR as a biomarker in patients with bladder cancer who underwent radical cystectomy should be further investigated in prospective studies involving larger patient cohorts and extended follow-up periods.

## Data Availability

The raw data supporting the conclusions of this article will be made available by the authors, without undue reservation.
